# Anatomical variations in the circle of Willis on magnetic resonance angiography in a south Trinidad population

**DOI:** 10.1093/bjro/tzad002

**Published:** 2023-12-12

**Authors:** Jason Diljohn, Fidel Rampersad, Paramanand Maharaj, Kristyn Parmesar

**Affiliations:** Radiology Unit, Department of Clinical Medical Sciences, The University of the West Indies, St. Augustine (UWI-STA), Trinidad, West Indies; Radiology Unit, Department of Clinical Medical Sciences, The University of the West Indies, St. Augustine (UWI-STA), Trinidad, West Indies; Radiology Unit, Department of Clinical Medical Sciences, The University of the West Indies, St. Augustine (UWI-STA), Trinidad, West Indies; Radiology Department, Arima General Hospital, Trinidad, West Indies

**Keywords:** neuroradiology, circle of Willis, anatomical variations circle of Willis, Trinidad, Trinidad and Tobago, stroke, magnetic resonance imaging, magnetic resonance angiography

## Abstract

**Objectives:**

This article seeks to determine the prevalence of a complete circle of Willis (CoW) and its common morphological variations in a south Trinidad population, while also investigating the influence of gender, age, and ethnicity on CoW morphology.

**Methods:**

A prospective, descriptive, cross-sectional study was done on the magnetic resonance images for consecutive patients who had a brain MRI/magnetic resonance angiography at a tertiary health institution in south Trinidad between October 2019 and September 2020. Patients with significant cerebrovascular disease and/or a history of prior neurosurgical intervention were excluded.

**Results:**

A complete CoW was seen in 24.3%, with more complete circles observed in younger participants (≤45 years) and Afro-Trinidadians. No gender predilection for a complete CoW was demonstrated. The most common variations in the anterior and posterior parts of the circle were a hypoplastic anterior communicating artery (8.6%, *n* = 13) and bilateral aplastic posterior communicating arteries (18.4%, *n* = 28), respectively.

**Conclusions:**

Significant variations exist in the CoW of a south Trinidad population with a frequency of complete in 24.3%, and more complete circles in younger patients and Afro-Trinidadians. Gender did not influence CoW morphology.

**Advances in knowledge:**

Structural abnormalities in the CoW may be linked to future incidence of cerebrovascular diseases and should therefore be communicated to the referring physician in the written radiology report. Knowledge of variant anatomy and its frequency for a particular populations is also required by neurosurgeons and neuro-interventional radiologists to help with preprocedural planning and to minimize complications.

## Introduction

### Background

The circle of Willis (CoW) is an important circulatory anastomosis responsible for maintaining a stable and redundant blood supply to the brain, thereby reducing the risk of ischaemia in the event of diminished cerebral blood flow. The CoW is found at the base of the brain and surrounds the pituitary stalk and optic chiasm.

The CoW is bounded anteriorly by the anterior communicating artery (ACoA) which connects both anterior cerebral arteries (ACAs). The ACAs course posterolaterally to reach the terminal segments of the internal carotid arteries (ICAs). At the point of connection between the ACA and the ICA, the lateral continuation of the ICA becomes the middle cerebral artery (MCA). The MCAs supply the lateral cerebral hemispheres excluding the superior parietal, inferior temporal, and occipital lobes, while the ACAs supply the frontal lobes in the midline and the superomedial parietal lobes.

The posterior communicating arteries (PCoAs) then join the MCAs to the posterior cerebral arteries (PCAs), which form the posterior limit of the CoW. Both PCAs unite to form the basilar artery at the base of the pons, which continues caudally anterior to the pons. The PCAs supply the occipital and inferior temporal lobes.

**Figure 1.**
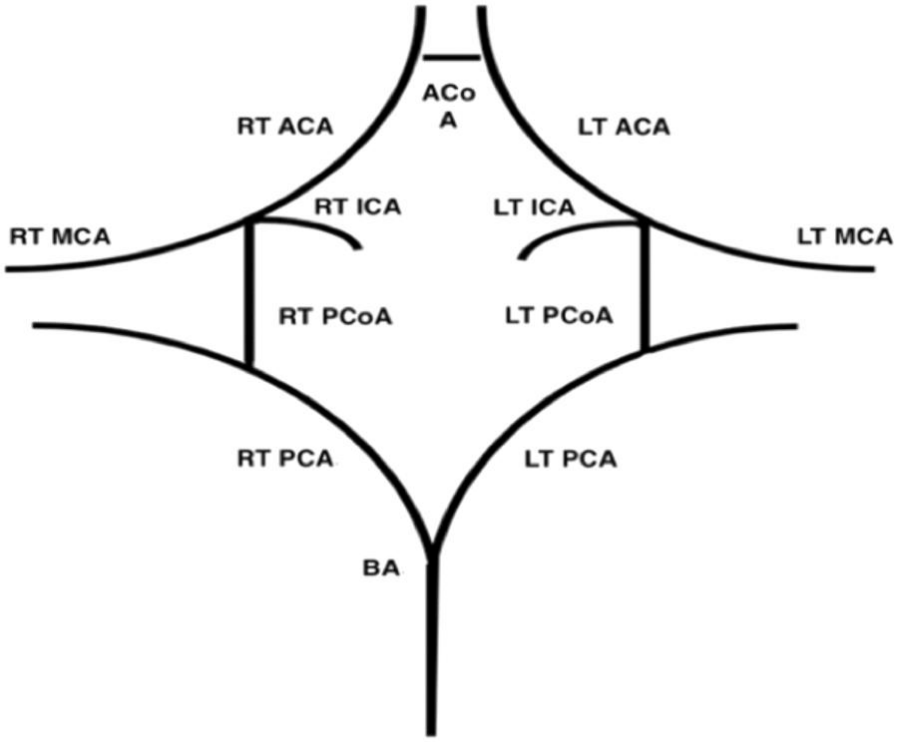
Schematic diagram demonstrating a complete circle of Willis. Abbreviations: RT = right; LT = left; ICA = internal carotid artery; MCA = middle cerebral artery; ACA = anterior cerebral artery; ACoA = anterior communicating artery; PCA = posterior cerebral artery; PCoA = posterior communicating artery; BA = basilar artery.

**Figure 2.**
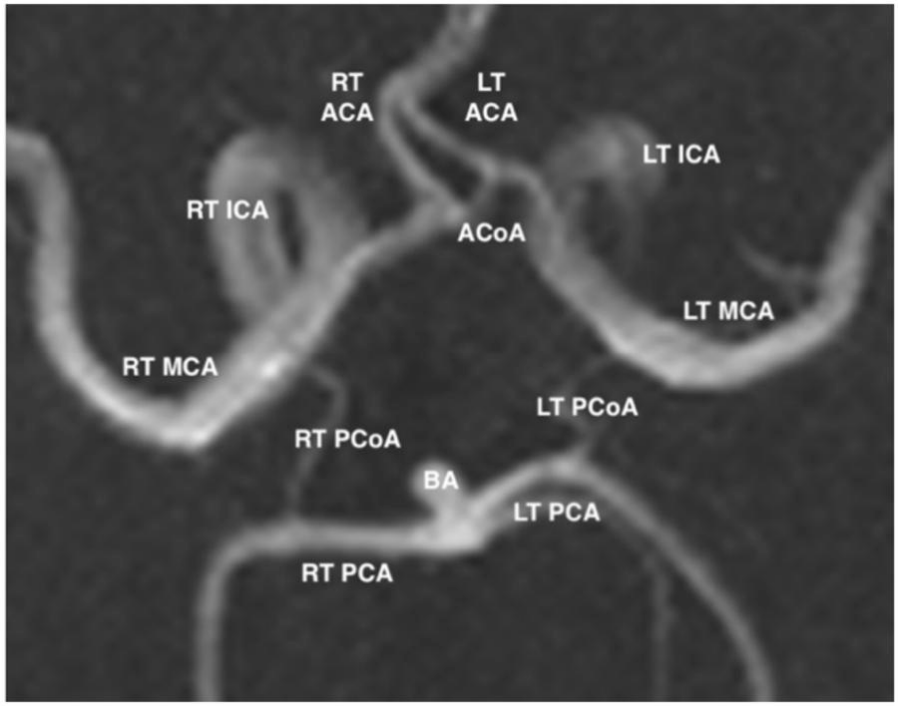
Axial maximum intensity projection magnetic resonance angiography image demonstrating a complete circle of Willis.

A schematic diagram demonstrating the “textbook” configuration of the CoW is depicted in Figure 1, while Figure 2 demonstrates a complete CoW obtained from a participant. While the above describes the “textbook” CoW configuration, this pattern is only present in 4.6%-72.2%.^1^

CoW anomalies described include aplasia, hypoplasia, and accessory vessels.^2–4^ The most common variant as described by Saikia et al.^5^ and Prasad et al.^6^ was PCoA hypoplasia in 20% and 26.2%, respectively, followed by foetal origin of the PCA in 13.8%, where the P1 segment of the PCA was smaller than the ipsilateral PCoA.

CoW examination can be done by various non-invasive methods including computed tomography angiography (CTA), magnetic resonance angiography (MRA), and transcranial doppler. The gold standard examination, however, is digital subtraction angiography (DSA), which provides a resolution unmatched to CTA and MRA. DSA is nevertheless an invasive procedure with inherent risks, including on-table ischaemic events, thromboembolism, and adverse reactions to iodinated contrast.

CTA acquires images quickly and provides excellent anatomy delineation. Disadvantages of CTA include exposure to ionizing radiation and possible adverse reactions related to iodinated contrast use.

MRA obtains images without the use of ionizing radiation and contrast medium. In MRA, flow-related enhancement is done by either time of flight or phase contrast imaging. MRA is 100% specific and 81.3%-100% sensitive for evaluating CoW variations.^5^

Structural abnormalities in the CoW may be linked to future incidence of cerebrovascular disease, intracranial aneurysms, and migraines. This study seeks to (1) establish the frequency of a complete CoW in a south Trinidad population, (2) determine the common anatomical variations in the CoW of this population, and (3) establish any relationship between ethnicity, age and gender, and CoW morphology.

## Methods

### Study design

A prospective, descriptive, cross-sectional study was done on magnetic resonance (MR) images for consecutive patients who had a brain MRI/MRA at a tertiary health institution in south Trinidad between October 1, 2019 and September 30, 2020.

### Study setting/site information

This study was conducted in south Trinidad at the Radiology Department of a 750-bed tertiary care centre with a catchment area of ∼600 000 persons (almost half of Trinidad’s population) and covering a surface area of ∼1/3 of the island.

Angiographic images were acquired on the same 1.5 T MR machine (Avantom Siemens, Germany) using a standard head coil and the time of flight method. Image parameters included (1) a repetition time/time to echo of 23/7.0, (2) 25-degree flip angle, (3) 0.7 mm slice thickness, (4) number of slices 44/slab, (5) number of slabs 4, (6) 25% slice overlap, (7) flow direction feet to head with 40 mm saturation at the head end, (8) 180 × 158 field of view, and (9) 256 matrix size. Scan images were reviewed on a CS2740 27-inch 4K ultra high definition in-plane switching monitor-ColorEdge EIZO, with a native resolution of 3840 × 2160 (16:9 aspect ratio), pixel density of 164 pixels per inch, and 350 cd/m^2^ brightness.

The population of Trinidad comprises individuals of various ethnic compositions, with the 2 major ethnic groups being Indo-Trinidadian (35.43%) and Afro-Trinidadian (34.22%). Minority ethnic groups include mixed (22.82%) and Caucasian (0.59%).^7^

Consecutive patients referred for brain MRI/MRA during the study time period were included with the exception of: (1) Patients with significant cerebrovascular disease, including suspected or confirmed major territorial ischaemic infarct and acute intracranial haemorrhage; (2) patients with a history of previous neurosurgical intervention; (3) patients with incidentally detected pathology including cerebral berry/saccular aneurysm, arteriovenous malformations and tumours; (4) repeat studies for the same patient; (5) unconscious/comatose patient; and (6) patients under the age of 18 years.

### Determination of sample size

The sample size was determined by using 2 methods. It was first calculated using the following formula (Daniel, 1999):


$$n=\frac{Z^{2}P\left(1-P\right)}{d^{2}}$$

where *n* = sample size,

*Z* = Z statistic for a level of confidence (confidence at 95%, *z* = 1.96),

*P* = expected prevalence or proportion (*P* is considered 0.3),

*d* = precision (in proportion of 1; if 7%, *d *=* *0.07).

The second approach entailed using a sample size comparative to that of similar studies. Similar studies were conducted by Maaly and Ismail^8^ in an Egyptian population, Shaikh and Sohail^9^ in an adult Pakistani population, and Saikia et al.^5^ in a northeastern Indian population comprising 180, 135, and 70 participants, respectively.

*Sample size calculation:*


$$\begin{array}{c} n=\frac{Z^{2}P\left(1-P\right)}{d^{2}}\\ n=\frac{1.96^{2}\left(0.3\right)*\left(1-0.3\right)}{\left(0.07^{2}\right)}\\ n=\frac{3.84\left(0.3\right)*\left(0.7\right)}{\left(0.0049\right)}\\ n=\frac{0.81}{0.0049}\\ n=165\;\text{participants}. \end{array}$$

Thus, a sample size of 165 participants was deemed adequate for this study.

### Data collection

After informed consent was obtained, the patient’s demographics (age, gender, and ethnicity [self-reported]), together with their past medical history, were attained and recorded in the participant information part of the data collection form by the MRI technologist.

### Data interpretation

Once completed, the MRI/MRA images were qualitatively assessed by the study co-observer (first observer), and findings recorded in Table 1 of the data collection form’s data analytic segment. These findings were later confirmed by the Principal Investigator (PI), a consultant Radiologist with 13 years’ experience (second observer), with significant interrater agreement (kappa = 0.7).

**Table 1.** Demographics of participants done by gender referred for head MRI/MRA at the San Fernando General Hospital between October 1, 2019 and September 30, 2020 (*n* = 152).

Demographic characteristic	All (152)	Male (43)	Female (109)
Age (*M*, *SD*)	45.69 (16.52)	49 (17)	44 (16)
Age group (*n*, %)			
18-25	22 (14.5)	6 (14.0)	16 (14.7)
26-35	29 (19.1)	3 (7.0)	26 (23.9)
36-45	22 (14.5)	8 (18.6)	14 (12.8)
46-55	34 (22.4)	10 (23.3)	24 (22.0)
56-65	27 (17.8)	9 (20.9)	18 (16.5)
66-75	13 (8.6)	6 (14.0)	7 (6.4)
76-85	5 (3.3)	1 (2.3)	4 (3.7)
Ethnicity (*n*, %)			
East Indian	81 (53.3)	25 (58.1)	56 (51.4)
African	64 (42.1)	17 (39.5)	47 (43.1)
Mixed	7 (4.6)	1 (2.3)	6 (5.5)

Abbreviations: M = mean; SD = standard deviation.

The anterior and posterior parts of the CoW were evaluated separately and classified utilizing a scheme resembling that used by Saikia et al.^5^ Permission to use this scheme was sought and obtained from Dr. Bishwajeet Saikia.

The A1 segment of the ACA was studied from its origin at the terminal segment of the ICA to its junction with the ACoA, while the A2 segment was examined along its path distal to the junction with the ACoA. The P1 segment of the PCA was studied from the basilar artery bifurcation to its junction with the PCoA, while the P2 segment was studied from its junction with the PCoA to its part in the perimesencephalic cistern.

Continuous vessels with a diameter ≥0.8 mm were considered present, while continuous vessels with a diameter <0.8 mm were regarded as hypoplastic. Vessels with non-continuous segments were considered absent, regardless of diameter. If the component vessels of both anterior and posterior parts were present and continuous, the CoW was considered complete. If both anterior and posterior parts were anomalous (hypoplastic or absent), the CoW was regarded as incomplete. The remaining CoWs with either a complete anterior or posterior part were considered partially complete.

### Statistical analysis

Statistical analysis was done using SPSS Statistics for Mac. The Chi-squared test was used to determine the impact of ethnicity, gender, and age on a complete CoW and its variant patterns. Data were demonstrated using tables, charts, and graphs.

## Results and observations

Data collection began on October 1, 2019 and concluded on September 30, 2020.

**Figure 3.**
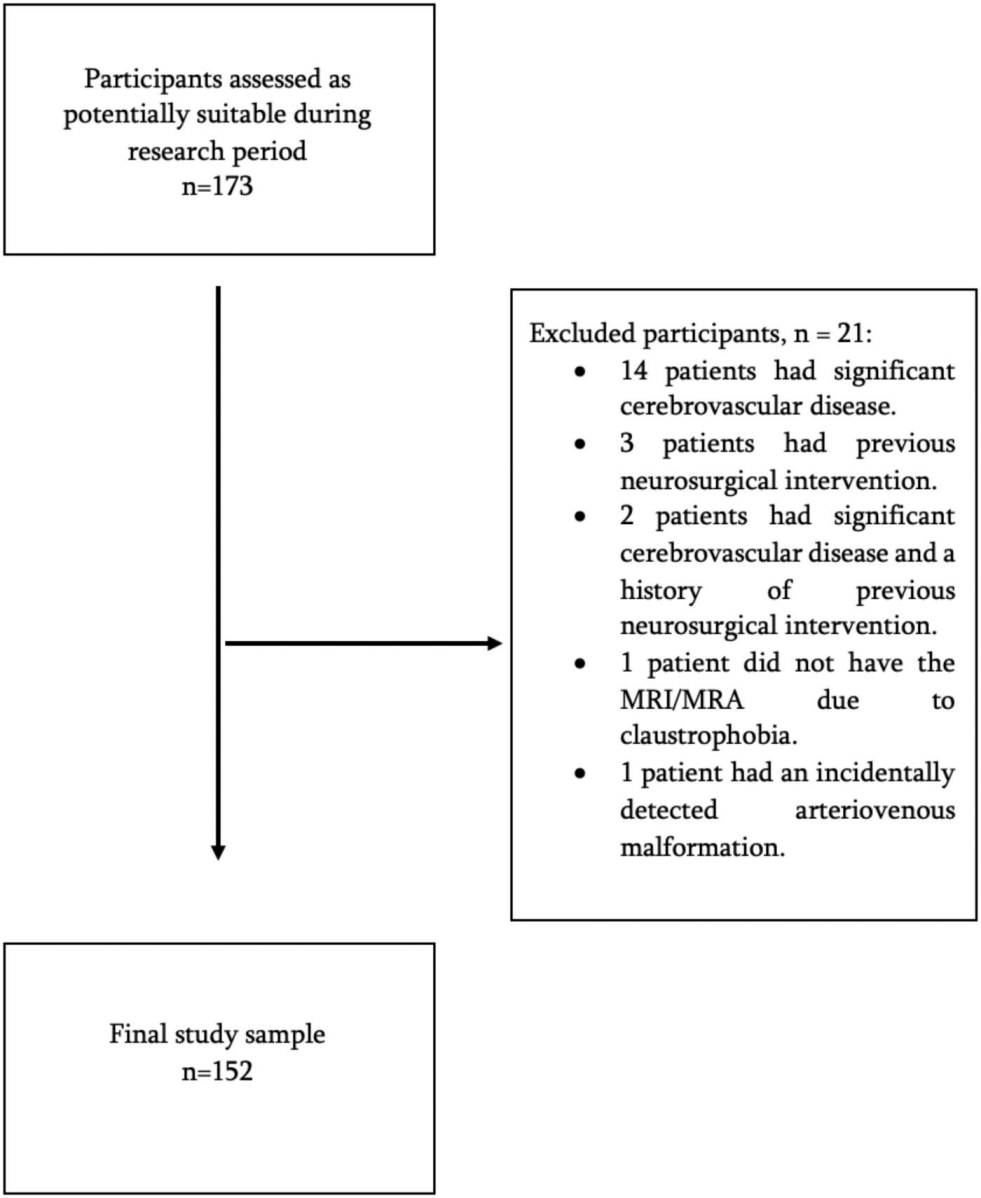
Flowchart demonstrating the study exclusion criteria and final study sample size.

A total of 173 participants had a head MRI/MRA during the research period. Of these 173, 21 participants were excluded, with 14 having significant cerebrovascular disease, 3 with previous neurosurgical intervention, and 2 with both significant cerebrovascular disease and previous neurosurgical intervention. Additionally, the MRI/MRA for one patient was not performed due to claustrophobia. Another case with an incidentally detected arteriovenous malformation was also excluded. A final study sample size of 152 was thus obtained. These results are depicted in Figure 3.

### Patient demographics

There were 152 participants: 28.3% (*n* = 43) male and 71.7% (*n* = 109) female. Participants’ ages ranged from 18 to 85 years. The mean participant age was 46.4 years and standard deviation 16.5. Most participants (22.4%, *n* = 34) were in the 46-55 age group, whilst the smallest number (3.3%, *n* = 5) in the 76-85 age group. The dominant ethnicity was Indo-Trinidadian with 53.3% (*n* = 81). Afro-Trinidadians accounted for 42.1% (*n* = 64) and mixed ethnicity 4.6% (*n* = 7). Table 1 shows the demographic characteristics of our study population.

### Frequency of a complete circle of Willis

**Figure 4.**
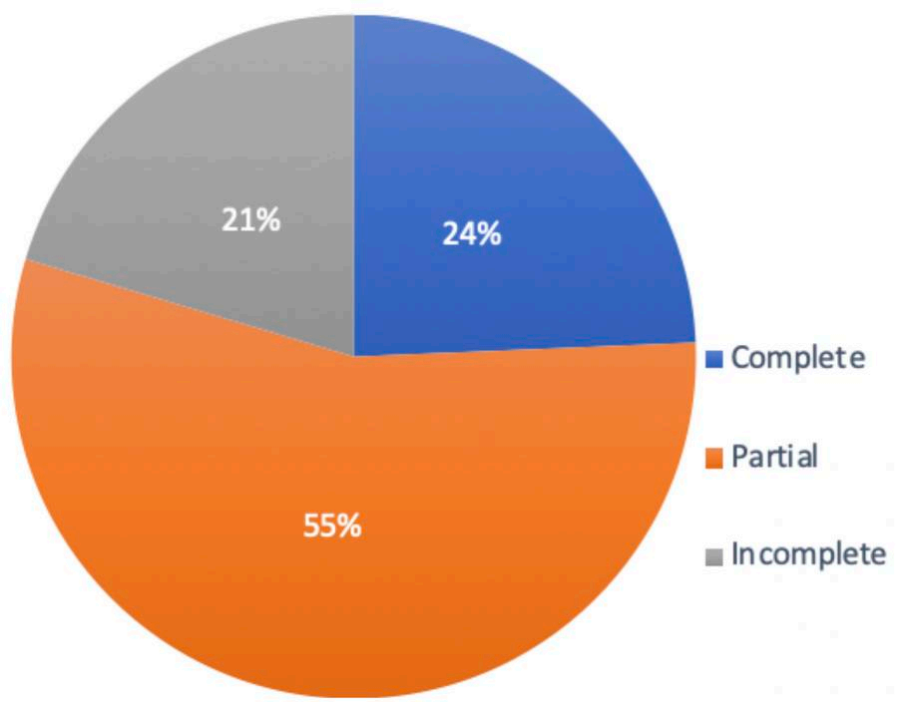
Pie chart demonstrating the incidence of a complete, partial, and incomplete circle of Willis in a south Trinidad population referred for head MRI/MRA at the San Fernando General Hospital between October 1, 2019 and September 30, 2020.

A complete CoW was seen in 37 participants (24.3%). Most cases (55.2%, *n* = 84), demonstrated a partial CoW, where either anterior or posterior part was anomalous. An incomplete CoW, where both anterior and posterior parts were deficient, was seen in 20.4% (*n* = 31). These results are shown in Figure 4.

### Variations in the anterior part of the circle of Willis

**Figure 5.**
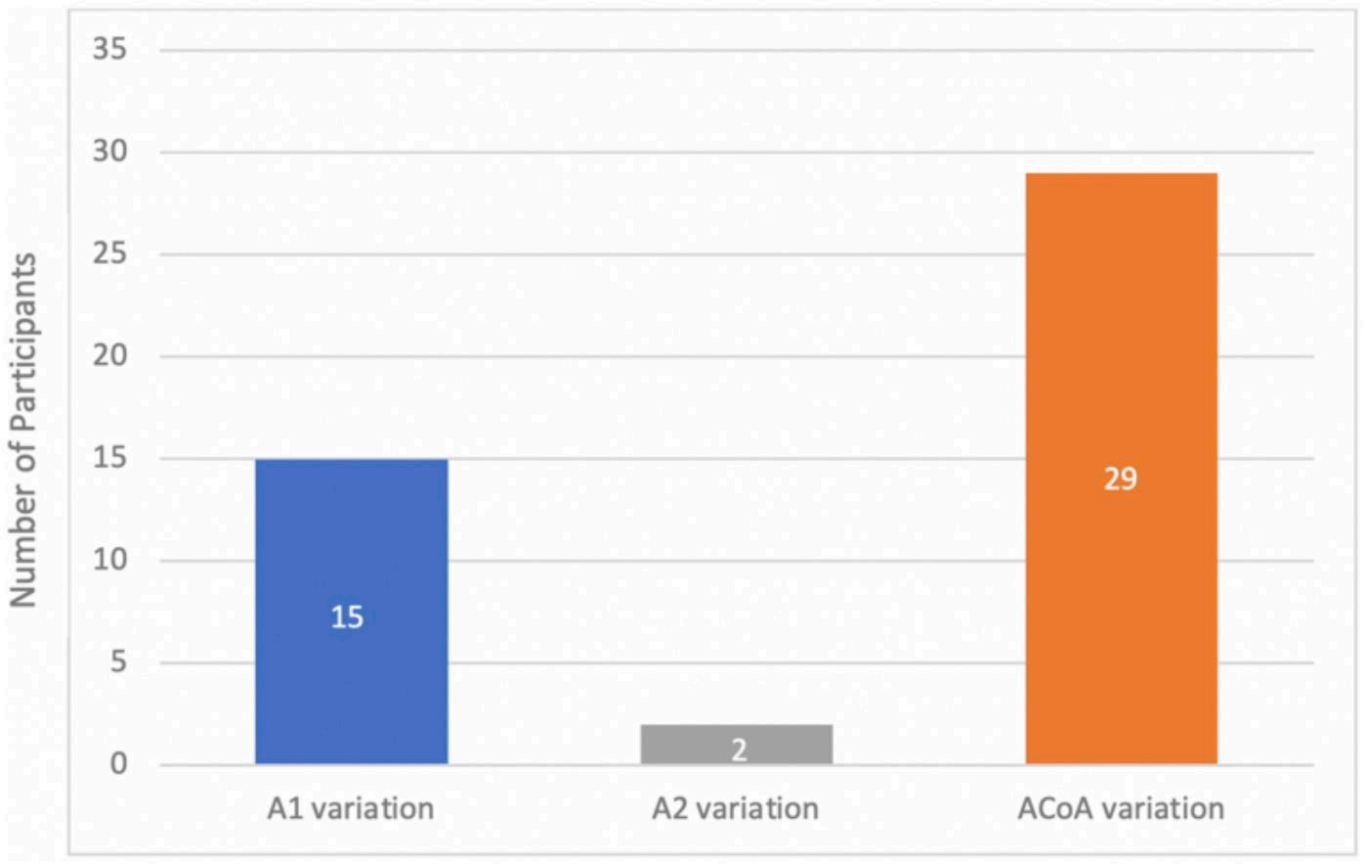
Bar graph demonstrating the frequencies of variations observed in the anterior part of the circle of Willis.

The anterior part of the CoW demonstrated variant anatomy in 46 cases (30.2%). The ACoA was most anomalous with 29 cases (19.1%), followed by the A1 segment of the ACA with 15 cases (9.9%). The A2 segment was only abnormal in 1.3% (*n* = 2). These results are depicted in Figure 5.

The most prevalent anterior circulation variant was a hypoplastic ACoA, seen in 13 cases (8.6%). Less common ACoA variations included a double ACoA (1.3%, *n* = 2), plexiform ACoA (0.7%, *n* = 1), and fenestrated ACoA (1.3%, *n* = 2). The next most frequent anterior circulation variant, seen in 11 cases (7.2%), was an absent A1 segment with both ACAs arising from the contralateral ICA. The frequencies of the other anterior CoW variants done according to gender, age, and ethnicity are demonstrated in Table 2. The anterior part of the CoW was complete in 106 cases (69.7%).

**Table 2.** Prevalence of variations in the anterior part of the circle of Willis done by gender, age, and ethnicity in a south Trinidad population referred for head MRI/MRA at the San Fernando General Hospital between October 1, 2019 and September 30, 2020 (*n* = 152).

	Anterior CoW variant												
	^a^ (11)	^b^ (4)	^c^ (2)	^d^ (0)	^e^ (0)	^f^ (0)	^g^ (4)	^h^ (7)	^i^ (13)	^j^ (2)	^k^ (1)	^l^ (2)	*P*-value
Gender (*n*, %)													.73
Male	4 (36.4)	2 (50.0)	0	0	0	0	1 (25)	2 (28.6)	4 (30.8)	1 (50.0)	1 (100)	0	
Female	7 (63.6)	2 (50.0)	2 (100)	0	0	0	3 (75)	5 (71.4)	9 (69.2)	1 (50.0)	0	2 (100)	
Age (*n*, %)													.84
18-45	3 (27.3)	1 (25.0)	1 (50.0)	0	0	0	2 (50)	1 (14.3)	6 (46.2)	1 (50.0)	0	1 (50)	
46-85	8 (72.7)	3 (75.0)	1 (50.0)	0	0	0	2 (50)	6 (85.7)	7 (53.8)	1 (50.0)	1 (100)	1 (50)	
Ethnicity (*n*, %)													.1
East Indian	7 (63.6)	3 (75.0)	1 (50.0)	0	0	0	4 (100)	1 (14.3)	9 (69.2)	2 (100)	0	2 (100)	
African	2 (27.3)	1 (25.0)	1 (50.0)	0	0	0	0	4 (57.1)	3 (23.1)	0	1 (100)	0	
Mixed ethnicity	1 (9.1)	0	0	0	0	0	0	2 (28.6)	1 (7.7)	0	0	0	

a Absent A1 segment with both ACAs arising from the opposite ICA.

b Hypoplastic A1 segment.

c Hypoplastic A2 segment.

d Fenestration of A1.

e Median artery of the corpus callosum.

f Fused ACA with absent ACoA.

g Absent ACoA with early origin of the callosomarginal branch.

h Absent ACoA.

i Hypoplastic ACoA.

j Double ACoA.

k Plexiform ACoA.

l Fenestrated ACoA.

### Variations in the posterior part of the circle of Willis

**Figure 6.**
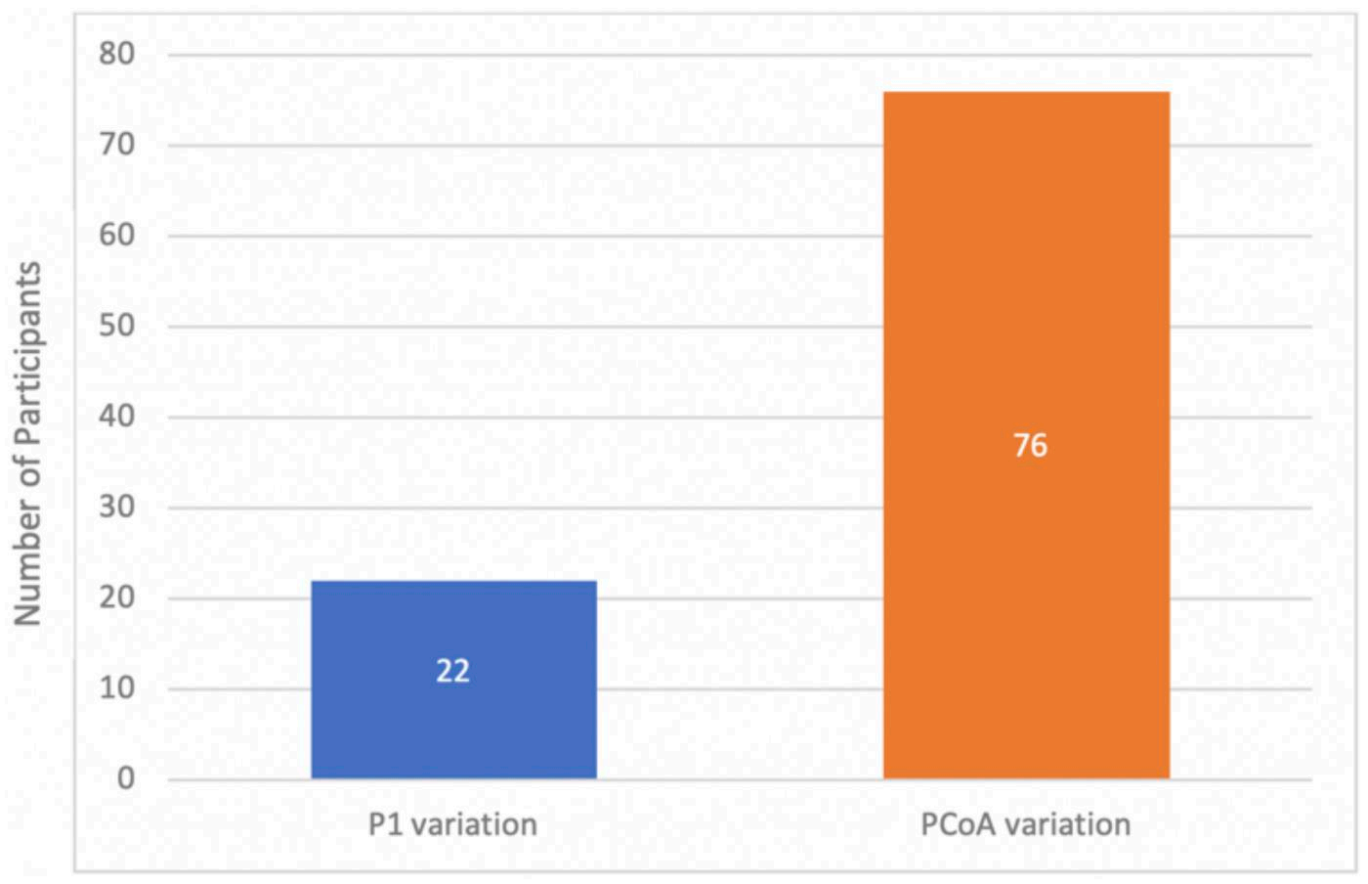
Bar graph showing the frequencies of variations observed in the posterior part of the circle of Willis.

Posterior CoW variant anatomy was found in 98 cases (64.5%). Most cases (50.0%, *n* = 76) demonstrated a deficient (hypoplastic or aplastic) PCoA. The next most anomalous vessel was the P1 segment of the PCA with 22 cases (14.5%). These results are shown in Figure 6.

The most prevalent posterior circulation variant was bilateral aplastic PCoAs, seen in 28 cases (18.4%), followed by unilateral aplastic PCoA, seen in 22 cases (14.5%). The frequencies of the other posterior circulation variations done according to gender, age, and ethnicity are demonstrated in Table 3. The posterior part of the CoW was complete in 54 cases (35.5%).

**Table 3.** Prevalence of variants in the posterior part of the circle of Willis done by gender, age, and ethnicity in a south Trinidad population referred for head MRI/MRA at the San Fernando General Hospital between October 1, 2019 and September 30, 2020 (*n* = 152).

	Posterior CoW variant								
	^a^ (7)	^b^ (1)	^c^ (12)	^d^ (2)	^e^ (18)	^f^ (8)	^g^ (22)	^h^ (28)	*P*-value
Gender (*n*, %)									.32
Male	1 (14.3)	0	7 (58.3)	0	3 (16.7)	3 (37.5)	5 (22.7)	9 (32.1)	
Female	6 (85.7)	1 (100)	5 (41.7)	2 (100)	15 (83.3)	5 (62.5)	17 (77.3)	19 (67.9)	
Age (*n*, %)									.12
18-45	2 (28.6)	0	5 (41.7)	0	6 (33.3)	2 (25.0)	10 (45.5)	7 (25.0)	
46-85	5 (71.4)	1 (100)	7 (58.3)	2 (100)	12 (66.7)	6 (75.0)	12 (54.5)	21 (75.0)	
Ethnicity									.01
East Indian	2 (28.6)	0	8 (66.7)	2 (100)	11 (61.1)	5 (62.5)	13 (59.1)	21 (75.0)	
African	3 (42.9)	1 (100)	3 (25.0)	0	5 (27.8)	3 (37.5)	9 (40.9)	6 (21.4)	
Mixed ethnicity	2 (28.6)	0	1 (8.3)	0	2 (11.1)	0	0	1 (1.9)	

a Hypoplastic P1 (U/L).

b Hypoplastic P1 (B/L).

c Absent P1 (U/L).

d Absent P1 (B/L).

e Hypoplastic PCoA (U/L).

f Hypoplastic PCoA (B/L).

g Aplastic PCoA (U/L).

h Aplastic PCoA (B/L).

Abbreviations: U/L = unilateral; B/L = bilateral.

### Variations in the circle of Willis with age

**Figure 7.**
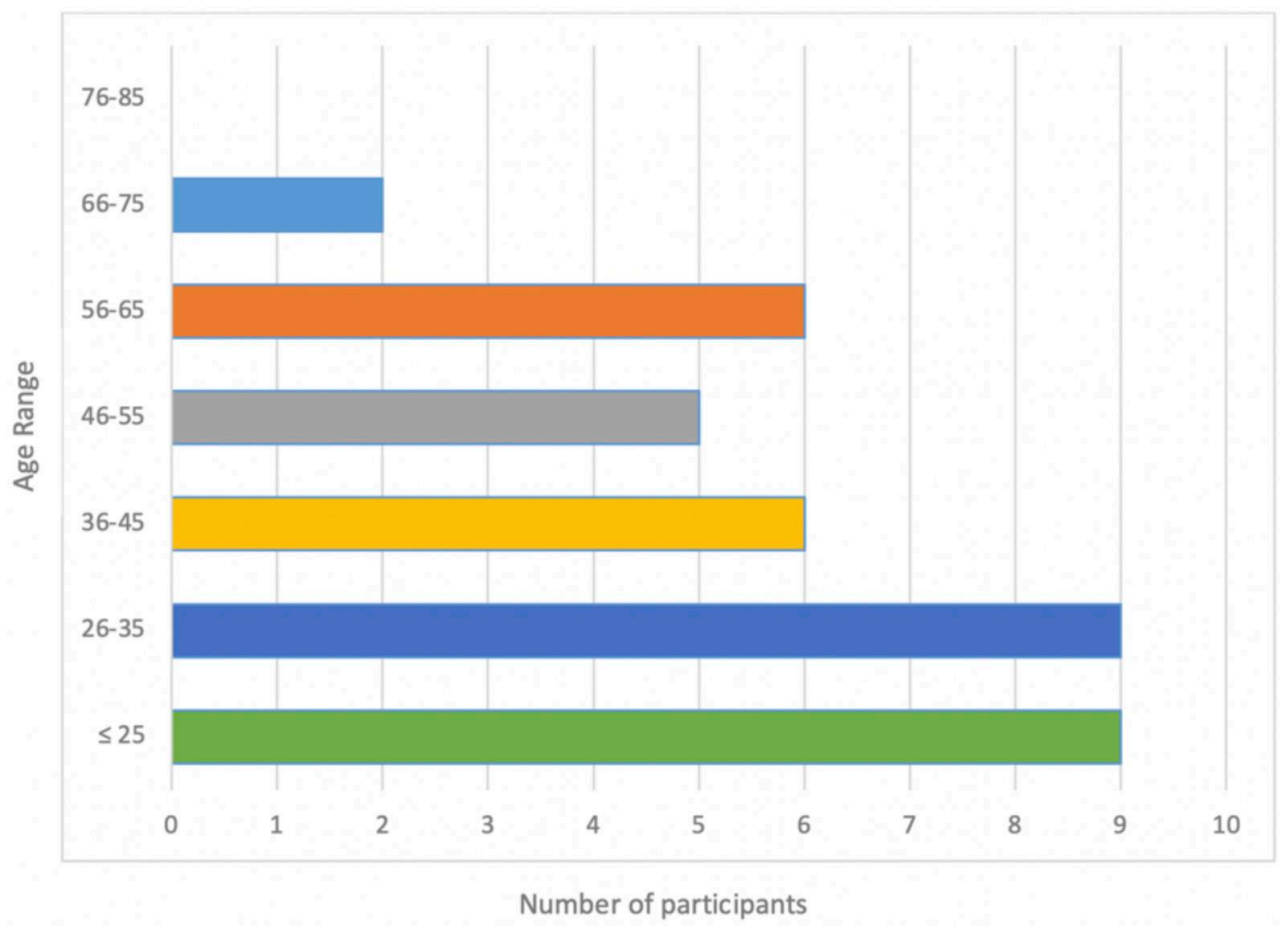
Horizontal bar graph showing the frequencies of complete circles observed in the different age groups.

There were more complete circles in participants ≤45 years (62.2%, *n* = 23) compared to participants ≥46 years (9.2%, *n* = 14). The largest number of complete circles (23.4%, *n* = 9) was seen in both the ≤25 and 26-35 age groups, while the smallest number of complete circles (5.4%, *n* = 2) was seen in the 66-75 age group. No complete circle was present in the 76-85 age group. These results are shown in Figure 7.

Participants ≤45 years demonstrated statistically significantly (*P* value = 0.01) less variation in the circle of Willis compared to the older population, with a partial CoW in 36.9% (*n* = 31) vs 63.1% (*n* = 53) and an incomplete CoW in 29% (*n* = 9) vs 71% (*n* = 22). Table 4 demonstrates the prevalence of a complete, partial, and incomplete CoW done by gender, age, and ethnicity.

**Table 4.** Prevalence of a complete, partial, and incomplete circle of Willis done by gender, age, and ethnicity in a south Trinidad population referred for head MRI/MRA at the San Fernando General Hospital between October 1, 2019 and September 30, 2020 (*n* = 152).

	Participants with a complete CoW (37)	Participants with a partial CoW (84)	Participants with an incomplete CoW (31)	*P*-value
Gender (*n*, %)				.28
Male	11 (29.7)	20 (23.8)	12 (38.7)	
Female	26 (70.3)	64 (76.2)	19 (61.3)	
Age (*n*, %)				.010
18-45	23 (62.2)	31 (36.9)	9 (29.0)	
46-85	14 (37.8)	53 (63.1)	22 (71.0)	
Ethnicity (*n*, %)				<.001
East Indian	9 (24.3)	53 (63.1)	19 (61.3)	
African	28 (75.7)	27 (32.1)	9 (29.0)	
Mixed ethnicity	0 (0.00)	4 (4.8)	3 (9.7)	

### Variations in the circle of Willis with gender

More complete circles were observed in females (70.3%, *n* = 26) (Table 4). This result was however not statistically significant (*P* value = .28). The most common anterior CoW variation in females was a hypoplastic ACoA, seen in 9 cases (69.2%), followed by an absent A1 segment with both ACAs arising from the contralateral ICA, in 7 cases (63.6%) (Table 2). In males, a dominant anterior CoW variant was not demonstrated with both hypoplastic ACoA and absent A1 segment with both ACAs arising from the contralateral ICA, accounting for 4 cases each. In the posterior part of the CoW, the most common variant observed in both genders was bilateral aplastic PCoAs, with 9 (32.1%) and 19 cases (67.9%) in males and females, respectively (Table 3).

### Variations in the circle of Willis with ethnicity

There was a statistically significant (*P* value < .001) greater number of complete circles in Afro-Trinidadians (75.7%, *n* = 28) compared to Indo-Trinidadians (24.3%, *n* = 9) (Table 4). The most common variant in the anterior circulation of Afro-Trinidadians was an absent ACoA seen in 4 cases (57.1%), and in Indo-Trinidadians was a hypoplastic ACoA with 9 cases (69.2%). In the posterior circulation, a unilateral aplastic PCoA was most prevalent in Afro-Trinidadians with 9 cases (40.9%), while bilateral aplastic PCoAs were most frequent in the Indo-Trinidadians with 21 cases (75.0%).

## Discussion

### Introduction

The CoW is an important circulatory anastomosis responsible for providing vital collateral circulation to the brain and neighbouring structures in the event of vascular compromise. Its effectiveness however relies on its completeness with slight variations having important clinical significance. A deficient CoW can increase the risk of ischaemic stroke due to reduced collated supply^10^^,^^11^ while faster flow through an abnormal configuration can increase wall shear stress and aneurysm formation.^11–14^ Individuals with a complete CoW also tend to have smaller infarcts and better neurological outcome after an ischaemic event.^15^^,^^16^

Since first described by English physician Sir Thomas Willis in 1664, numerous studies have been done describing the variant anatomy of the CoW. Today, cross-sectional imaging allows for non-invasive examination of the CoW with high degrees of specificity and sensitivity. At our institution, the cerebral circulation is examined non-invasively using CTA and MRA.

### Influence of ethnicity, gender, and age on CoW morphology

The frequency of a complete CoW varies between populations ranging from 22.2% in an adult Pakistani population^9^ to 46.7% in an Egyptian population.^8^ Other frequencies obtained were 24.28% in a northeastern Indian population,^5^ 35.4% in a Nepalese population,^6^ 34.50% in a Nigerian population,^17^ and 69.57% in a Malawian population.^18^ Our sample, which comprised predominantly East Indians (54.9%) showed a complete CoW in 24.3% and an incomplete/partial CoW in 75.7%. The 24.3% frequency of a complete CoW seen in our East Indian dominant population is similar to that seen in the adult Pakistani population (22.2%) and the northeastern Indian population (24.28%). However, when separated our Afro-Trinidadian population demonstrated statistically significant (*P* value < .001) more complete circles than our Indo-Trinidadian population, with a prevalence of complete in 75.7% (*n* = 28), similar to that seen in the black Malawian population (69.6%). As far as we know, this study is the first to demonstrate frequency variations for a complete CoW between ethnic groups in a single multiethnic population.

This result may be correlated with those found in a paper published by Mungrue et al.,^19^ which demonstrated more strokes in Indo-Trinidadians. The Indo-Trinidadian group in our study had fewer complete CoWs than the Afro-Trinidadian population, thereby possibly increasing their likelihood for a major cerebrovascular event. Further study in this area is warranted to draw definitive conclusions.

CoW completeness between genders varies, with several studies demonstrating a female predominance,^20–22^ some a male predominance,^23^^,^^24^ and others equal frequencies.^25^ In our study, there was no statistically significant difference (*P* value = 0.12) between our male and female populations.

In our population, more complete circles (62.2%, *n* = 23) were seen in patients <46 years than patients ≥46 years (37.8%, *n* = 14). These results were statistically significant (*P* value = 0.01). Similar results were documented by Krabbe-Hartkamp et al.,^20^ Naveen et al.,^22^ Eaton et al.,^25^ and Zaninovich et al. who all demonstrated an inverse relationship between age and completeness,^26^ a relationship now well established in the literature. The exact mechanism behind this change has not yet been determined, but has been suggested to occur secondary to a combination of atherosclerosis and other lifestyles diseases, including diabetes mellitus and hypertension,^27–29^ which cause vessel occlusion over time and radiological absence, but not anatomical absence, a theory supported by more complete CoWs in cadaveric studies.

### Variation in the anterior and posterior parts of the circle of Willis

It is widely recognized today that greater variation exists in the posterior part of the CoW, with higher rates of completeness in the anterior part. In our study, variation in the posterior part of the CoW was seen in 98 cases (64.5%), while anterior circulation variation was only demonstrated in 46 cases (30.3%). Frequencies of the morphological variations in the anterior and posterior parts by gender, age, and ethnicity are demonstrated in Tables 2 and 3, respectively.

**Figure 8.**
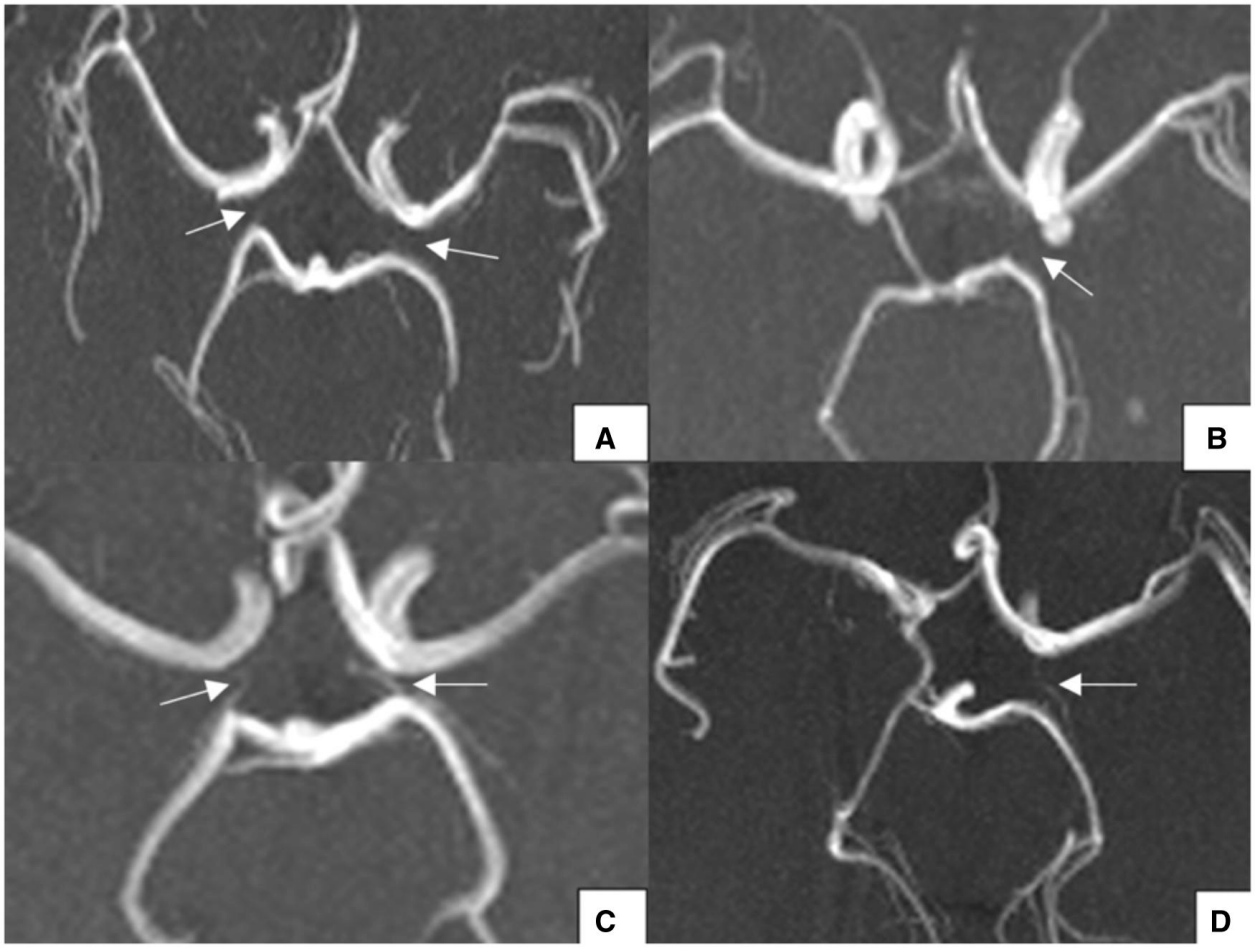
Axial maximum intensity projection magnetic resonance angiography images demonstrating variants seen in the posterior part of the circle of Willis in our population: (A) bilateral aplastic PCoA; (B) unilateral aplastic left PCoA; (C) bilateral hypoplastic PCoA and (D) unilateral hypoplastic left PCoA. Abbreviation: PCoA = posterior communicating artery.

In the posterior part, 76 cases (50%) demonstrated variation in the PCoA, while 22 cases (14.5%) showed variation in the P1 segment of the PCA. Aplasia accounted for the most common PCoA variation, with 28 cases (18.4%) having bilateral aplastic PCoAs and 22 cases (14.5%) having a unilateral aplastic PCoA. Figure 8A and B shows axial maximum intensity project (MIP) images from 2 participants demonstrating bilateral aplastic PCoAs and a unilateral aplastic left PCoA, respectively. Similar findings to those seen in our study were demonstrated by Zaninovich et al.^26^ who recorded bilateral aplastic PCoAs as the dominant variant in 17.1%, followed by unilateral PCoA aplasia.

PCoA hypoplasia was next most common with 26 cases (17.1%). There were 18 cases (11.8%) with unilateral PCoA hypoplasia and 8 cases (5.3%) with bilateral PCoA hypoplasia. Figure 8C and D shows axial MIP images from 2 participants demonstrating bilateral hypoplastic PCoAs and a unilateral aplastic left PCoA, respectively.

The P1 segment of the PCA demonstrated less variation than the PCoA (14.5%, *n* = 22). Absent P1 segments were seen in 14 cases (9.2%) and a hypoplastic P1 segment was seen in 8 cases (5.3%).

The variations demonstrated in the posterior part of the CoW in our population are not surprising as all recent studies have demonstrated greater variation in the posterior circulation, with PCoA variations being most common.^3^^,^^5^^,^^8^^,^^18^^,^^25^^,^^26^ Milenkovic et al.^30^ and Van Overbeeke et al.^31^ have proposed that such variation may be due to the increased vascular demand of the rapidly developing occipital lobes *in utero*.

In the anterior part of the CoW, the ACoA was most anomalous with 19.1% (*n* = 29). Aplasia and hypoplasia were the most common ACoA variants with 24.1% (*n* = 7) and 44.8% (*n* = 13), respectively. This was similar to studies published by Prasad et al.^6^ and Shaikh and Sohail^9^ who both recorded the ACoA as most anomalous in the anterior circulation.

**Figure 9.**
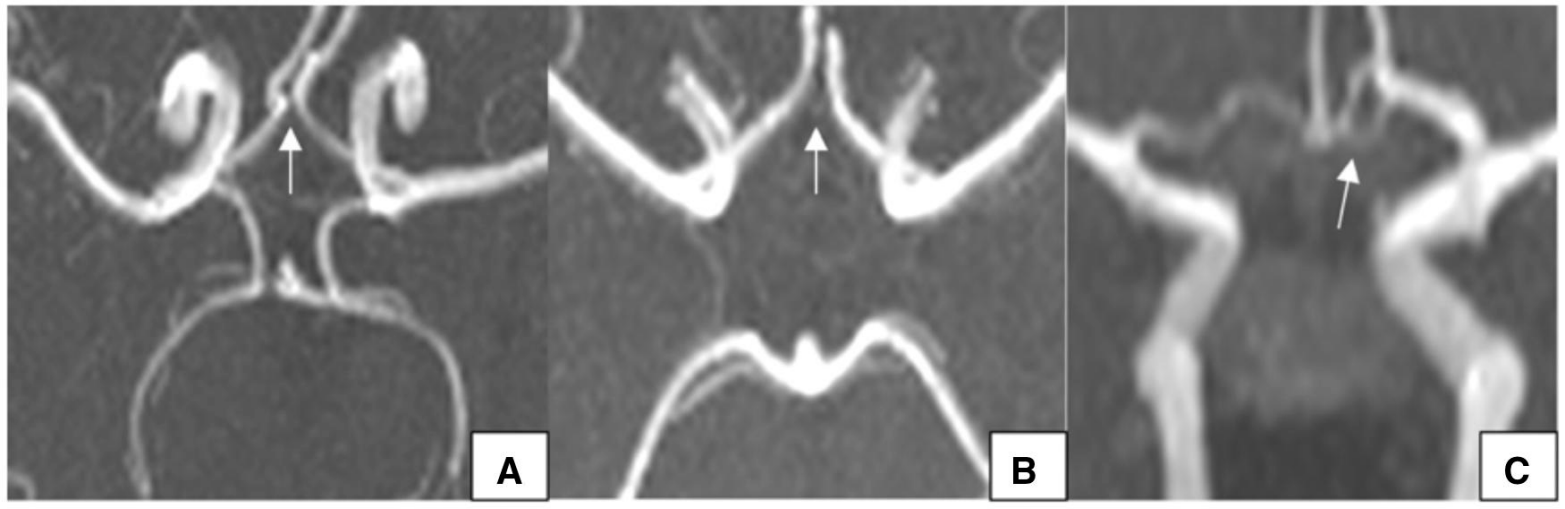
Axial (A and B) and coronal (C) maximum intensity projection magnetic resonance angiography images demonstrating anterior communicating artery variations seen in our study: (A) absent ACoA; (B) hypoplastic ACoA; and (C) fenestrated ACoA. Abbreviation: ACoA = anterior communicating artery.

While the ACoA functions as an important collateral pathway in ICA stenosis/occlusion, it is often documented as anomalous in imaging studies. This finding is, however, not supported by examination in surgical procedures, which document very low frequencies of ACoA aplasia and hypoplasia.^6^ The reason for such frequent ACoA variation in imaging studies may, therefore, be due to poor visualization because of its small size and technical limitations inherent to the imaging modality, rather than anatomical absence. Double, plexiform, and fenestrated ACoA were less common ACoA variants observed, with 2 (1.3%), 1 (0.7%), and 2 cases (1.3%), respectively. Figure 9 demonstrates some variations seen in the ACoA of our population.

**Figure 10.**
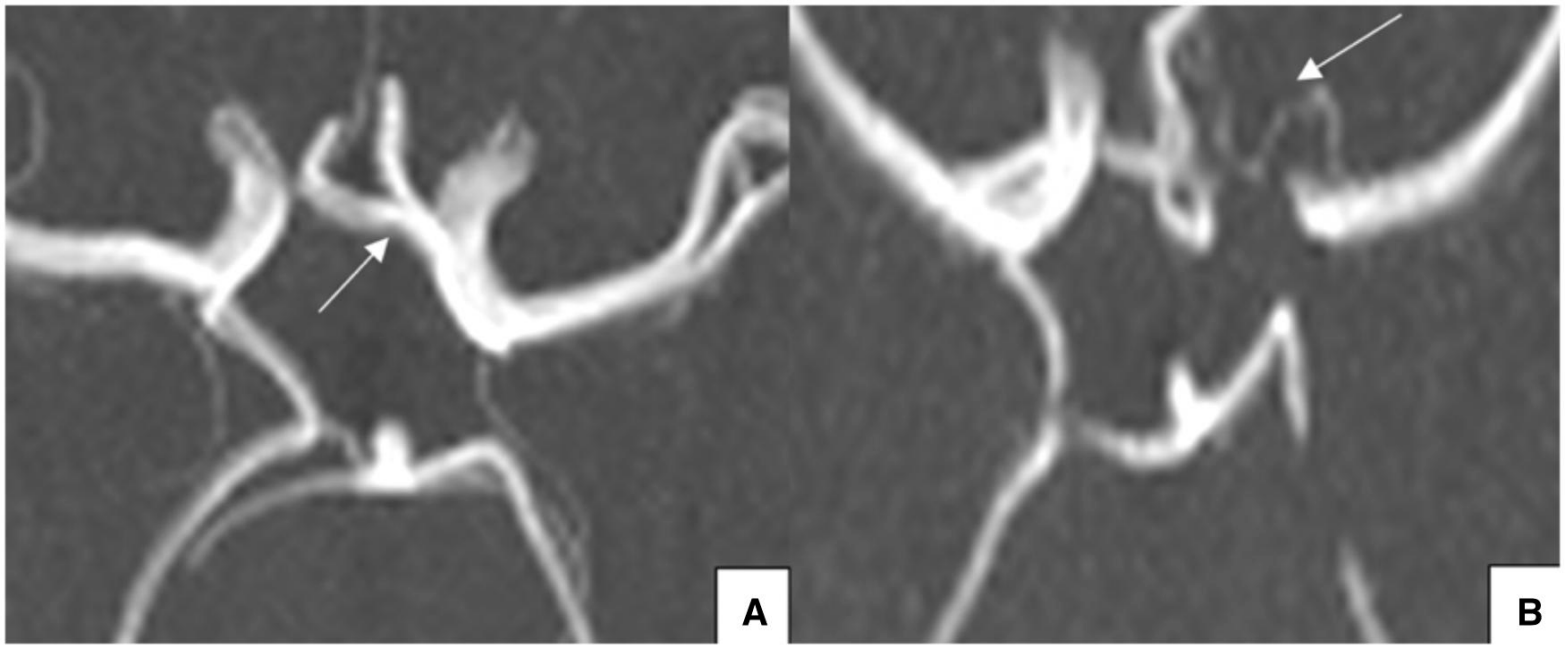
Axial maximum intensity projection magnetic resonance angiography images demonstrating variations seen in the A1 segment of the ACA in our population: (A) absent A1 segment of the right ACA with both ACAs arising from the opposite ICA (white arrow); (B) hypoplastic A1 segment of the left ACA. Abbreviation: ACA = anterior cerebral artery.

Variation in the A1 segment of the ACA was seen less frequently (9.9%, *n* = 15), with most cases demonstrating an absent A1 segment with both ACAs arising from the opposite ICA (73.3%, *n* = 11). There was no statistically significant difference (*P* value = 0.12) between ACA variation and age which may suggest that variation with large calibre vessels, such as the ACA, might actually be present from birth rather than from occlusion over time^30^ as compared to the more frequently seen smaller vessel variants. Figure 10 demonstrates the variations seen in the A1 segment of our population.

In a post mortem analysis of the CoW in an Indian population, a complete circle was seen in 77.8%.^32^ Such high prevalence of a complete circle has not been demonstrated by any imaging study and may be due to the fact that non-patent, anatomically complete vessels are deemed present in post mortem analyses, while only functional vessels are considered present in imaging studies. Imaging studies should thus be considered superior to post mortem analyses since, non-patent, anatomically present vessels do not provide vital collateral supply in the event of reduced cerebral blood flow.

### Influence of environmental and genetic factors

According to Forgo et al.,^33^ the influence of environmental and genetic factors on CoW variants still remains unclear but may have some influence. One study demonstrated more PCoA variations in families hence suggesting a heritable pattern.^28^ Other authors have suggested a genetic background,^27^^,^^34^^,^^35^ while stochastic and environmental factors^36^ may also be possible, such as preeclampsia and prematurity.^37^

Malamateniou et al.^37^ described more complete circles in preterm at term infants, and less CoW variations in these infants, compared to term infants. Interestingly also, preterm infants born at <30 weeks (*n* = 22, 50%) demonstrated more complete circles and less vascular variations compared to preterm infants born after 30 weeks (*n* = 19, 36.8%). A hypothesis suggested by the author for these findings was possible vascular remodelling in the cerebral circulation of this vulnerable population, which serves as a protective mechanism to the developing brain. Similar to our study, the most common morphological variant recorded by Malamateniou et al.^37^ was an absent PCoA, accounting for 30%. No statistically significant difference between gender and anatomical variations was also demonstrated by Malamateniou et al.

## Study limitations

Our imaging method, MRA, was our primary study limitation. DSA is considered the gold standard examination for CoW morphology, but is not available at our institution. Technical factors associated with MRA include motion artefact due to long MR imaging times, which could degrade image quality and interpretation. Additionally, spurious flow voids, could result in misclassification of smaller vessels as aplastic rather than hypoplastic.

The definition of a complete CoW was not consistent among authors, with some authors excluding both hypoplastic and aplastic vessels from their definition of a complete CoW, and others including hypoplastic vessels in their definition of a complete CoW. In our study, a complete CoW excluded both hypoplastic and aplastic vessels. Hypoplastic vessels were defined as vessels with a diameter <0.8 mm, while aplastic vessels were defined as vessels with non-continuous segments regardless of vessel diameter.

The vessel diameter used to categorize hypoplasia was also not consistent among authors with some classifying hypoplasia as <0.8 mm, and others using 1.0 mm as the lower limit of normal. In our study, we used <0.8 mm to represent hypoplasia, similar to other studies.^20^^,^^38–40^

Although this study was done at a tertiary institution in south Trinidad catering for approximately half of Trinidad and Tobago’s population, it was restricted to the southern part of the island, which may have influenced the results obtain. Multicentre studies can be done in the future to overcome this bias.

## Opportunities for future research/recommendations

Our study comprised a relatively small cohort of 152 cases. A larger sample is recommended for future studies, as this will strengthening the validly of these findings and allow for generalization of results.

The study population comprised inherently sick patients referred to a tertiary hospital for head MRI/MRA. Using a healthy population might potentially produce different results and should be considered for future research.

Atherosclerosis and lifestyle diseases paralleling ageing have been postulated to affect CoW morphology and hence may have influenced the results demonstrated in our study. Future studies should consider the effect of lifestyle diseases, including diabetes mellitus and hypertension, on CoW morphology.

Jin et al.^23^ demonstrated a greater number of aneurysms in patients with a family history of stroke, while Sánchez van Kammen et al.^28^ showed a familial association with PCoA variations. It is recommended that further study of CoW morphology in families be conducted based on these observations.

The effects of environmental factors, including prematurity and pre-eclampsia, have been documented to affect cerebral vascular anatomy^37^^,^^41^ and should be considered in future studies evaluating CoW morphology.

## Conclusion

In this study, CoW anatomy assessed by MRA showed significant variation from the “textbook” configuration, which can increase the risk of cerebrovascular disease, including ischaemic stroke and cerebral aneurysm formation, due to altered cerebral haemodynamics.

An inverse relationship was demonstrated between a complete CoW and increasing age, a relationship now well established in literature and speculated to occur secondary to atherosclerotic occlusion and lifestyle diseases. Ethnicity also affected CoW morphology with Afro-Trinidadians having more complete circles than Indo-Trinidadians. There was no gender predilection for a complete CoW in our study.

Similar to other populations worldwide, there was greater variation in the posterior circulation of our population, with bilateral aplastic PCoAs (18.4%) being the most common variant. The most common variant seen in the anterior part of the CoW in our population was a hypoplastic ACoA (8.6%).

Moreover, this study provides an important reference for CoW morphology in a Trinidadian population and further reinforces the importance of a complete CoW and its relevance in providing vital collateral circulation, thereby reducing the morbidity and mortality associated with cerebral vascular compromise.
